# The Dutch SCORE-based risk charts seriously underestimate the risk of cardiovascular disease

**DOI:** 10.1007/s12471-016-0927-2

**Published:** 2016-12-09

**Authors:** H. T. Jørstad, S. M. Boekholdt, N. J. Wareham, K. T. Khaw, R. J. G. Peters

**Affiliations:** 1 Department of Cardiology, Academic Medical Center, University of Amsterdam, Amsterdam, The Netherlands; 2 MRC Epidemiology Unit, Institute of Metabolic Science, Addenbrooke’s Hospital, Cambridge, UK; 3 Department of Public Health and Primary Care, Institute of Public Health, University of Cambridge, Cambridge, UK

**Keywords:** Cardiovascular disease, Risk assessment, Risk prediction, Epidemiology

## Abstract

**Introduction:**

Dutch cardiovascular disease (CVD) prevention guidelines recommend the use of modified SCORE risk charts to estimate 10-year risk of fatal and nonfatal CVD (myocardial infarction, cerebrovascular disease and congestive heart failure). This combined risk is derived from the SCORE mortality risk using multipliers. These multipliers have been shown to underestimate overall CVD risk. We aimed to compare the current Dutch risk charts with charts that estimate a broader range of clinically relevant CVD using updated multipliers.

**Methods:**

We constructed new risk charts for 10-year CVD using updated, recently published multipliers from the EPIC-Norfolk study, based on ratios of fatal CVD to clinically relevant CVD (fatal plus nonfatal CVD requiring hospitalisation for ischaemic heart disease, cardiac failure, cerebrovascular disease, peripheral artery disease, and aortic aneurysm). Our primary outcome was the proportion of the three risk categories, i. e. ‘high risk’ (>20% 10-year risk), ‘intermediate risk’ (10–19%) and ‘low risk’ (<10%) in the new risk charts as compared with the current risk charts.

**Results:**

Applying the updated fatal CVD/clinical CVD multipliers led to a marked increase in the high-risk categories (109 (27%) vs. 244 (61%), (*p* < 0.001)), an absolute increase of 229%. Similarly, the number of low-risk categories decreased (190 (48%) vs. 81 (20%) (*p* < 0.001)).

**Conclusion:**

The current Dutch risk charts seriously underestimate the risk of clinical CVD, even in the first 10 years. Even when analyses are restricted to CVD events that required hospitalisation, true 10-year risks are more than double the currently estimated risks. Future guidelines may be revised to reflect these findings.

## Introduction

Current multidisciplinary guidelines on cardiovascular disease (CVD) risk management (CVRM) in the Netherlands recommend using a modified version of the Systematic COronary Risk Evaluation (SCORE) to estimate 10-year risk of fatal and nonfatal CVD [1]. When exceeding a predefined threshold (≥20%), it is recommended to initiate or intensify preventive measures. The original SCORE chart and algorithm on which the modified, current version is based is the ‘low-risk’ SCORE [2], which estimates 10-year risk of fatal CVD only. Using data from two different national cohorts [1, 3, 4], multipliers have been calculated to convert the risk of 10-year fatal CVD to the risk of 10-year fatal and nonfatal CVD, including first nonfatal hospitalisations for myocardial infarction (MI), cerebrovascular disease and congestive heart failure (CHF). These multipliers are 5× the SCORE predicted fatal CVD risk for individuals aged 35–45 years, 4× for individuals aged 45–65 years, and 3× for individuals aged >65 years. Overall risk is presented in the charts, and coded by colour [1].

These multipliers have not been validated in other large population-based studies, and include only three clinical manifestations of nonfatal CVD. Recently, we published an analysis of the ratios of fatal CVD to total CVD in the European Prospective Investigation of Cancer and Nutrition-Norfolk (EPIC-Norfolk), a large prospective population-based cohort in the UK [5]. In this study, we observed a complex relationship between fatal CVD and a broad range of clinically relevant (requiring hospitalisation) CVD (fatal and nonfatal CVD including ischaemic heart disease (IHD), cerebrovascular disease, CHF, peripheral arterial disease and aortic aneurysm), with decreasing fatal CVD to clinical CVD ratios with increasing age, and with greater ratios for women in all age groups, suggesting that such ratios are highly age- and sex-dependent.

Therefore, in our current study, we applied these new ratios to the original low-risk SCORE charts to design a new, updated risk chart, and compared the updated risk chart with the current risk chart.

## Methods

### Source population

We used data from the EPIC-Norfolk prospective population study, a cohort of 25,639 men and women aged 39–79 residing in the county of Norfolk in the UK. Details of the study have been described elsewhere [6]. In brief, between 1993 and 1997, 77,630 adults were invited from general practices to participate in the study. Of these, 25,639 (33%) provided signed informed consent for study participation and attended a baseline health assessment. During this visit, data were collected on medical history, drug use, anthropometrics, blood pressure, and laboratory measures. The participants’ National Health Service number was used to identify hospitalisations through the East Norfolk Health Authority database. Vital status for all EPIC-Norfolk participants was obtained through death certification at the Office for National Statistics. The underlying cause of death or hospital admission was coded by trained nosologists according to the International Classification of Diseases (ICD), Tenth Revision. The EPIC-Norfolk study was approved by the Norfolk Local Research Ethics Committee and complies with the Declaration of Helsinki [6]. We report results for follow-up to 31 March 2008, a mean follow-up of 11 years.

### Study design

To compare the effect of applying different ratios to the SCORE charts, we constructed a new, updated risk chart using the ratios found in the EPIC-Norfolk study. These ratios and the fatal and nonfatal CVD rates on which they are based have recently been published [5].

In our analysis, fatal CVD was defined as death where CVD was reported as the underlying cause of death on the death certificate. Clinically manifest CVD was defined as fatal CVD plus hospitalisation with CVD as the underlying cause, including five different presentations of CVD (IHD, CHF, cerebrovascular disease, peripheral artery disease, and aortic aneurysm), hereafter referred to as ‘*CVD-updated’*. The current risk charts include fatal CVD and nonfatal CVD from three manifestations of CVD (MI, cerebrovascular disease, and CHF), hereafter referred to as ‘*CVD-current*’.

The SCORE risk charts consist of three levels of risk: green (<10% risk of 10-year CVD), yellow (10–19% risk of 10-year CVD), and red (≥20% risk of 10-year CVD), which have consequences for the initiation or intensification of risk management strategies. We aimed to quantify the effect of applying the updated multipliers for clinically manifest CVD (*CVD-updated*) to the current risk charts (based on *CVD-current*) by comparing the number of patient categories within the three risk levels in the current risk charts with the number of patient categories in the updated risk charts.

Only EPIC-Norfolk participants who did not report a history of MI or cerebrovascular disease at the baseline health assessment were included in our analysis. We excluded individuals with diabetes mellitus, as diabetes mellitus is not included as a variable in the SCORE algorithm.

As the multipliers in the Dutch guideline are based on fatal CVD and nonfatal CVD including only MI, cerebrovascular disease and CHF, we performed a sensitivity analysis. In this analysis, we calculated a second set of ratios of fatal CVD to fatal and nonfatal CVD using only IHD, cerebrovascular disease and CHF for the nonfatal CVD outcomes in individuals aged 39–70 years.

### Statistical methods

Baseline characteristics were summarised separately for men and women, using numbers and percentages for categorical data, means, 95% confidence intervals (CI) and standard deviations (SD) for continuous data with a normal distribution, and median and interquartile range for continuous variables with a non-normal distribution. Ten-year rates of fatal CVD and clinically relevant CVD were estimated using the Kaplan-Meier method. Ratios of fatal CVD to clinically relevant CVD (*CVD-updated*) were calculated for the total population and in age groups (40–50, 50–55, 55–60, 60–65, 65–70), for men and women separately. In individuals with a 10-year risk of fatal CVD >0%, we applied the ratios from our previous study (men 39–50 years 11.7, 50–55 years 9.9, 55–60 years 9.5, 65–70 years, 6.9; women 39–50 years 28.5, 50–55 years 19.6, 55–60 years 17.8, 60–65 years 9.1, 65–70 years 6.4) to calculate risk of clinically relevant CVD (*CVD-updated*) [5]. As no ratio could be applied to risk levels of 0%, these were marked as ‘<1%’ in the risk charts. Risks were coloured in accordance with the current risk charts: green <10%; yellow 10–19%; red ≥20%. In accordance with the current guidelines, risk levels higher than 50% were described as ‘>50%’. To estimate the effects of adding the broader range of clinically manifest CVD to the risk charts, we quantified the number of risk categories by summarising numbers of coloured squares in the current risk charts and our updated CVD charts, which were compared using Fisher’s exact tests. Statistical analyses were performed in SPSS 22 and STATA 13.

## Results

The selected EPIC-Norfolk study population consisted of 24,014 men (43.8%) and women (56.2%) without a history of MI, cerebrovascular disease or diabetes mellitus. The population characteristics are presented in Table 1. Mean age was 58.8 (SD 9.3) years, and 11.8% were current smokers. Mean body mass index, total cholesterol and LDL-cholesterol were 26.3 kg/m^2^ (SD 3.9), 6.2 mmol/l (SD 1.2) and 4.0 mmol/l (SD 1.1), respectively, which is slightly above the levels recommended in primary prevention settings. The rate of 10-year fatal CVD was 3.9% (900 events); the rate of clinically relevant CVD was 21.2% (4978 fatal or nonfatal events).

**Table 1:** Population characteristics of EPIC-Norfolk participants

Population characteristics	Total	Male	Female
(*n* = 24,014)	(*n* = 24,014)	(*n* = 10,509)	(*n* = 13,505)
Age, years	58.8 ± 9.3	59.0 ± 9.3	58.7 ± 9.3
Body mass index, kg/m^2^	26.3 ± 3.9	26.4 ± 3.3	26.2 ± 4.3
Current smokers	2836 (11.8)	1297 (12.3)	1539 (11.4)
Systolic blood pressure, mm Hg	135.2 ± 18.3	137.1 ± 17.5	133.7 ± 18.8
Diastolic blood pressure, mm Hg	82.4 ± 11.2	84.4 ± 11.1	80.9 ± 11.1
Total cholesterol, mmol/l	6.2 ± 1.2	6.0 ± 1.1	6.3 ± 1.1
LDL cholesterol, mmol/l	4.0 ± 1.0	3.9 ± 1.0	4.0 ± 1.1
HDL cholesterol, mmol/l	1.4 ± 0.4	1.2 ± 0.3	1.6 ± 0.4

Data are presented as number (percentage), mean ± standard deviation, or median (interquartile range)

*LDL* low-density lipoprotein, *HDL* high-density lipoprotein

**Fig. 1:**
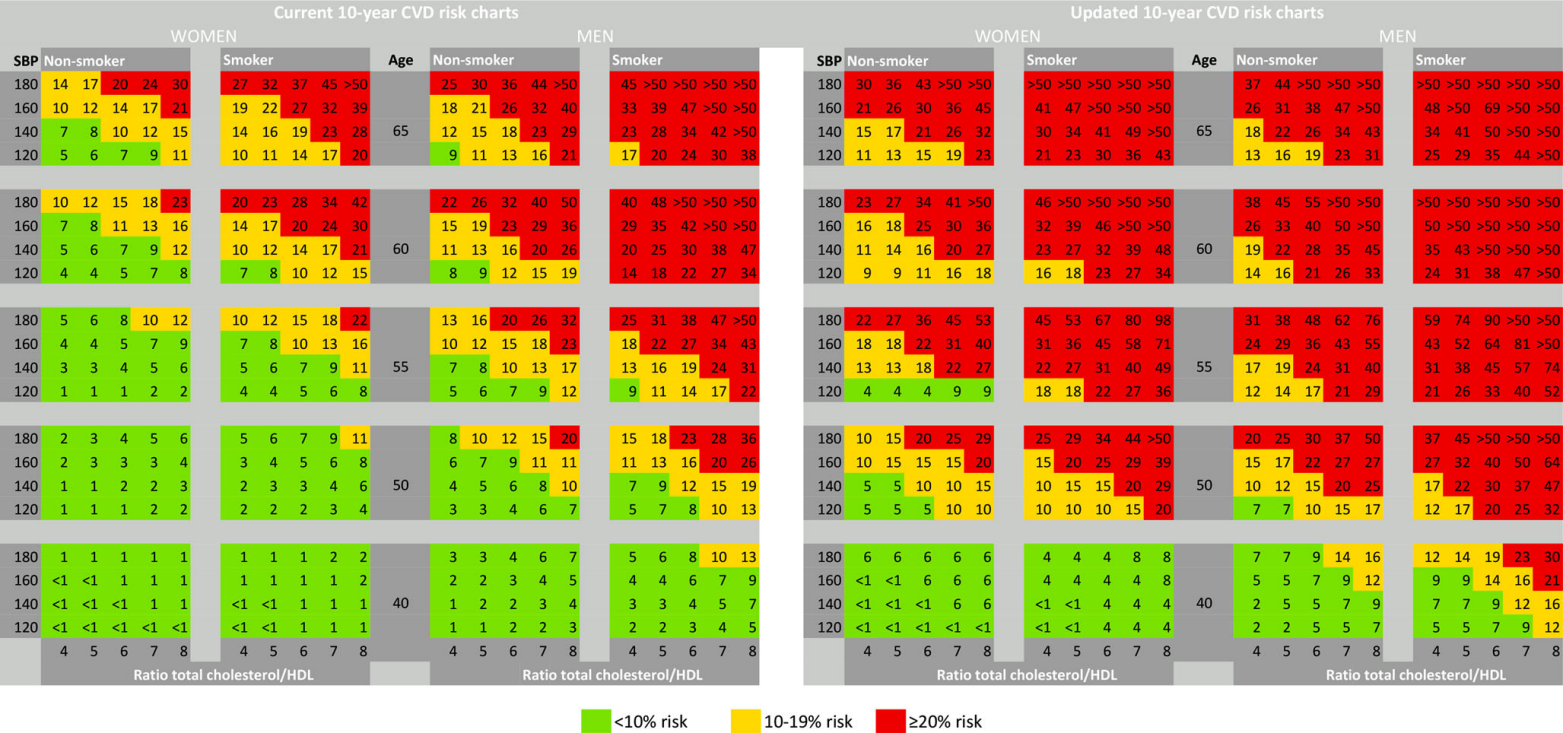
Risk charts of 10-year risk of CVD (Left panel: 10-year risk of fatal CVD and nonfatal myocardial infarction, cerebrovascular disease, and congestive heart failure, as in the current Dutch preventive guidelines. Right panel: Updated risk chart for 10-year risk of clinically relevant CVD (any fatal or nonfatal CVD, including ischaemic heart disease, cerebrovascular accident, congestive heart failure, peripheral artery disease, and aortic aneurysm). Numbers are % 10-year risk. *CVD* cardiovascular disease)

**Fig. 2:**
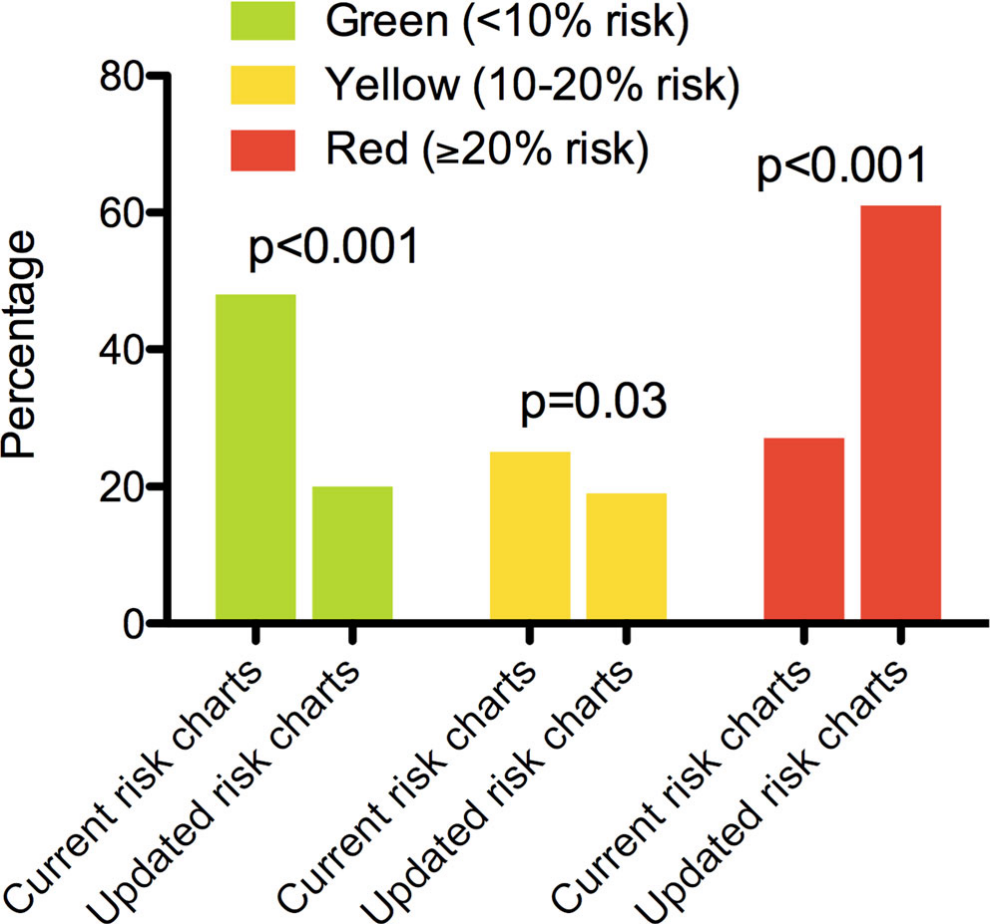
Risk stratification according to colour in the current risk charts and in the updated CVD risk charts (*CVD* cardiovascular disease)

Overall, the multipliers were 3.7 times higher when using the outcomes of *CVD-updated* (5 clinical manifestations) as compared with *CVD-current* (3 clinical manifestations); in women (4.9×) higher than in men (2.4×). Illustrating this, Fig. 1 shows the current risk charts and the updated CVD risk charts based on the multipliers from *CVD-updated*. Whereas the current charts contain in total 109 (27%) red squares, i. e. signifying a combination of risk factors amounting to a 10-year fatal and nonfatal CVD risk of ≥20%, deemed as ‘high risk’, this number increased to 244 (61%, *p* < 0.001) when accounting for clinically relevant CVD, an absolute increase of 229%. Similarly, the numbers of patient categories at ‘low risk’ (<10%) decreased from 190 (48%) to 81 (20%) (*p* < 0.001) when accounting for clinically relevant CVD (Fig. 2).

**Fig. 3:**
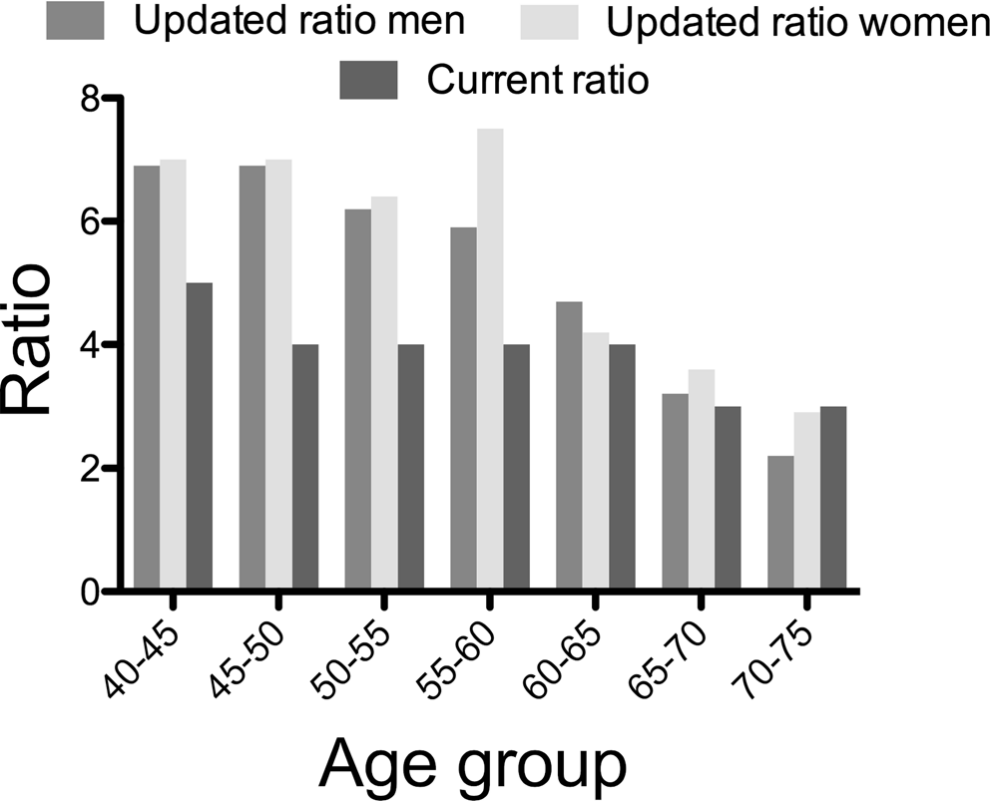
Current score chart ratios compared with EPIC-Norfolk ratios for CVD mortality and CVD mortality plus IHD, cerebrovascular disease and CHF (*CVD* cardiovascular disease, *IHD* ischaemic heart disease, *CHF* congestive heart failure)

In the sensitivity analysis, we limited the number of outcome events to three instead of five clinical manifestations of CVD (fatal CVD and nonfatal IHD, cerebrovascular disease, and CHF). In total, there were 1844 events when using this outcome definition, amounting to a cumulative event rate of 9.5% (95% CI 9.1–9.9). When calculating the ratios of fatal CVD to this selection of clinical events, these ratios remained markedly higher in the younger age groups (Table 2) as compared with the current multipliers (Fig. 3). In individuals aged 60 years or older, the multipliers were more in agreement with the currently used multipliers.

**Table 2:** Cumulative 10-year CV mortality and CV mortality and nonfatal ischaemic heart disease/cerebrovascular disease/CHF by sex and age in EPIC-Norfolk

Sex	Age group		10-year CV mortality			10-year CV mortality and nonfatal IHD/stroke/CHF			Ratio
		*N*	*n*	KM rate	95%CI	*n*	KM rate	95%CI	
Male	39–50	2219	15	0.7	(0.4–1.1)	104	4.8	(4.0–5.8)	6.9
	50–55	1780	26	1.5	(1.0–2.2)	160	9.3	(8.1–10.8)	6.2
	55–60	1637	34	2.1	(1.5–3.0)	190	12.3	(10.7–14.0)	5.9
	60–65	1633	67	4.2	(3.4–5.4)	299	19.7	(17.8–21.8)	4.7
	65–70	1622	127	8.3	(7.0–9.8)	384	26.3	(24.1–28.6)	3.2
	*Total*	8891	269	3.1	(2.8–3.5)	1137	13.5	(12.8–14.3)	4.4
Female	39–50	3061	5	0.2	(0.07–0.4)	43	1.4	(1.1–1.9)	7.0
	50–55	2333	11	0.5	(0.3–0.9)	71	3.2	(2.5–4.0)	6.4
	55–60	2129	17	0.8	(0.5–1.3)	122	6.0	(5.1–7.1)	7.5
	60–65	2014	43	2.2	(1.6–2.9)	175	9.3	(8.0–10.1)	4.2
	65–70	1995	86	4.5	(3.6–5.5)	296	16.4	(14.7–18.2)	3.6
	*Total*	11,206	162	1.4	(1.2–1.7)	707	6.4	(6.0–6.9)	4.6

CVD mortality is death from a cardiovascular disease. CVD mortality and nonfatal IHD/cerebrovascular disease/CHF is all fatal cardiovascular disease or nonfatal IHD/ cerebrovascular disease/CHF requiring hospitalisation. Cumulative event rates were calculated using the Kaplan-Meier method

Ratio is the ratio of CVD mortality/ CV mortality and nonfatal IHD/cerebrovascular disease/CHF of the Kaplan-Meier estimates

*CVD* cardiovascular disease, *CI* confidence interval, *KM* Kaplan-Meier, *IHD* ischaemic heart disease, *CHF* congestive heart failure

## Discussion

Our analysis shows that the current risk charts as recommended by the Dutch CVRM guideline seriously underestimate the risk of nonfatal CVD, even in the first 10 years. Applying multipliers to calculate nonfatal CVD from fatal CVD using a greater number of clinical outcome events (5 versus 3) leads to a drastically higher risk estimation, especially in young individuals, and in women more than in men. When the multipliers are applied to the current risk charts, an increase is observed of 229% in the proportion at ‘high risk’. Consequently, the current focus on a limited number of vascular territories for nonfatal events in risk stratification potentially leaves large numbers of individuals untreated, even though their risk of CVD is substantial.

The definition and choice of CVD events is essential in any study investigating the relationship between fatal CVD and different manifestations of nonfatal CVD. We believe that for adequate counselling on CVD preventive strategies, all outcomes that are relevant to patients should be included. In our analysis, we only included events requiring hospitalisation, while milder CVD, i. e. peripheral artery disease or CHF not requiring hospitalisation, were not included. While these manifestations do not require hospitalisation, they are relevant to patients, providers of healthcare, policy makers, and insurance companies. Furthermore, in recent decades, CVD mortality has shown a decline relative to CVD morbidity, and the burden of total CVD is likely to increase [7, 8]. Consequently, even our adjusted multipliers are likely to underestimate true risk.

Individual lifetime risks of fatal and nonfatal CVD, instead of 10-year risk, could potentially be more relevant to patients and caregivers. Lifetime CVD mortality has been shown to be markedly higher than 10-year risk [9]. However, 10-year risk estimation is a practical approach in assessing risk, and helps caregivers evaluate whether preventive therapies should be initiated or may be postponed, dependent on future reassessment.

The type of first nonfatal CVD event could potentially influence preventive strategies. The majority of first nonfatal events or hospitalisations in our population were caused by ischaemic CVD (77.6%), including IHD, ischaemic cerebrovascular disease and peripheral arterial disease [5]. A recent analysis in the same population has shown that different risk factors have different impacts on atherosclerotic CVD manifestations [10]. Therefore, in individuals with a high risk-factor burden, or in which a sequential approach to risk factor optimisation is desired, this could potentially aid the choice of initial therapies (i. e. aggressive LDL-lowering to prevent coronary artery disease, intensified blood pressure control to prevent peripheral artery disease and cerebrovascular disease), taking into account each individuals’ clinical circumstances [11, 12].

Several factors play a role when interpreting the different versions of the SCORE risk charts. In a recent paper, we found that SCORE slightly overestimates mortality risk (10–39% in men, 21–82% in women) in the UK [13]. This was most prominent for fatal coronary heart disease (overestimation of 61%) as compared with fatal non-coronary heart disease (a slight underestimation of 13%). With decreasing case fatality rates over time, the ratios between mortality and morbidity are expected to increase. Therefore, several factors simultaneously contribute to over- and under-estimation of risk within the SCORE algorithm. In addition, landmark trials have reported varying ratios of fatal to nonfatal CVD, [14, 15] and it has been hypothesised that these differences reflect diagnostic differences (such as ascertainment and diagnostic thresholds) rather than underlying disease differences [16]. The risk charts in their original form were published in 2003, based on 12 large European cohorts, with inclusion periods ranging from 1967 to 1991. Since the inclusion started in the earliest cohorts of the original SCORE population (1967), therapeutic strategies have changed considerably. While a comparison of the effect of these changes is difficult to quantify across the respective cohorts, such changes in therapeutic strategies certainly influence the total burden of CVD and the rates of fatal and nonfatal CVD. As a consequence of these changing event rates, several countries initially classified as high-risk countries, including both the UK and the Netherlands, have now been reclassified as low-risk countries [3, 13, 17]. Due to these temporal trends, we believe that findings in the most recent cohort, i. e. EPIC Norfolk, may reflect current event rates most accurately.

Furthermore, there are important differences in the population characteristics in the original SCORE cohort, as compared with the EPIC-Norfolk, MORGEN and ERGO cohorts (Tables 3 and 4), which should be taken into account when interpreting our results. The SCORE cohort had more men as compared with the later cohorts, and the prevalence of smoking was considerably higher. Also, mean blood pressure was higher in the ERGO cohort as compared with the other cohorts, and ERGO only included individuals ≥55 years of age.

**Table 3:** Baseline characteristics of the original SCORE cohort and EPIC-Norfolk

	SCORE cohort^2^ (*n* = 205,178)		EPIC-Norfolk (*n* = 24,014)	
Inclusion years	1967–1991		1993–1997	
Age, range	45–64		39–70	
	Men	Women	Men	Women
*n*, (%)	117,098 (57)	88,080 (43)	10,509 (44)	13,505 (56)
Age, mean	nr	nr	59.0 (±9.3)	58.7 (±9.3)
Smoking, %	51	27	12.3	11.4
TC, mmol/l	6.1	6.0	6.0 (±1.1)	6.3 (±1.1)
HDL, mmol/l	1.3	1.2	1.2 (±0.4)	1.6 (±0.4)
SBP, mm Hg	139	133	137.1 (±17.5)	133.7 (±18.8)
BMI, kg/m^2^	nr	nr	26.4 (±3.3)	26.2 (±4.3)

Data extracted from original publications (reference as number in superscript). Numbers are presented as mean and (±SD) (when available), range, or percentage

*nr* not reported, *TC* total cholesterol, *HDL* high-density lipoprotein cholesterol, *SBP* systolic blood pressure, *BMI* body mass index

**Table 4:** Baseline characteristics of the MORGEN and ERGO cohorts

	MORGEN^3^ (*n* = 32,887)		ERGO^4^ (*n* = 6045)	
Inclusion years	1987–1997		1990–1993	
Age, range	37.5–62.5		≥55	
	Men	Women	Men	Women
*n*, (%)	15,457 (47)	17,430 (53)	2,287 (38)	3,758 (62)
Age, mean	46 (±6.5)	49 (±6.6)	67.9 (±8.3)	69.7 (±9.4)
Smoking, %	38	37	31.0	18.5
TC, mmol/l	5.7 (±1.1)	5.7 (±1.1)	6.3 (±1.2)	6.8 (±1.2)
HDL, mmol/l	1.1 (nr)	1.1 (nr)	1.2 (±0.3)	1.4 (±0.4)
SBP, mm Hg	126 (±15.9)	121 (±17)	139 (±22)	140 (±22)
BMI, kg/m^2^	26.0 (±3.4)	25.6 (±4.3)	25.6 (±3.0)	26.7 (±4.0)

Data extracted from original publications (reference as number in superscript). Numbers are presented as mean and (±SD) (when available), range, or percentage

*nr* not reported, *TC* total cholesterol, *HDL* high-density lipoprotein cholesterol, *SBP* systolic blood pressure, *BMI* body mass index

### Strengths and limitations

There are several strengths to our study. First, we performed our analysis in a large, population-based cohort with long-term follow-up. Detailed information on fatal and nonfatal outcomes and hospitalisation was available, and we were able to analyse event rates in large subgroups based on age and sex. Second, the EPIC-Norfolk cohort is comparable with a representative UK sample for anthropometric variables, blood pressure and serum lipids [6]. It should however be noted that the population in the Norfolk area is healthier than the general UK population with a standardised mortality ratio of 0.94 (source: Office for National Statistics). Third, a large number of outcome events were available, which were coded by trained nosologists according to the relevant ICD codes, based on the underlying cause of death or hospital admission. Previous validation studies in this cohort indicated high specificity of such case ascertainment [18].

Some aspects of our study warrant consideration. First, the EPIC-Norfolk population study is a UK study. Ideally, our analysis should have been performed in a contemporary Dutch cohort. This is not available. However, both countries are currently categorised as low-risk countries, justifying the use of the same SCORE algorithms and risk charts in both populations. Second, CVD not requiring hospitalisation, including ‘mild’ peripheral artery disease, ‘mild’ heart failure or stable angina pectoris, was not included in our analysis. Not including these ‘milder’ manifestations of CVD leads to an underestimation of the total risk of CVD. Third, CVD other than IHD and cerebrovascular disease was not recorded at baseline in our cohort. Therefore, we cannot exclude that some of the study participants included in our analysis were already treated in the setting for secondary prevention instead of primary prevention, making them ineligible for risk stratification using the SCORE charts. However, these individuals were similarly not excluded in the original SCORE cohorts [2].

### Conclusion

In conclusion, the 10-year risk of clinically relevant CVD in an individual is significantly greater than is currently estimated based on the current Dutch SCORE charts recommended by the CVRM guideline. Even when analyses are restricted to CVD events that require hospitalisation, true 10-year risks are more than double the currently estimated risks. Caution is advised when using the current risk charts, especially in young individuals, as a low risk according to the current risk charts may not reflect a low risk of clinically relevant CVD. Future guidelines may need to be revised to reflect these findings.
